# *N-*Glycan Modification of a Recombinant Protein via Coexpression of Human Glycosyltransferases in Silkworm Pupae

**DOI:** 10.1038/s41598-017-01630-6

**Published:** 2017-05-03

**Authors:** Tatsuya Kato, Natsumi Kako, Kotaro Kikuta, Takatsugu Miyazaki, Sachiko Kondo, Hirokazu Yagi, Koichi Kato, Enoch Y. Park

**Affiliations:** 1 Laboratory of Biotechnology, Department of Applied Biological Chemistry, Faculty of Agriculture, Shizuoka University, 836 Ohya, Suruga-ku, Shizuoka 422-8529 Japan; 2 Laboratory of Biotechnology, Research Institute of Green Science and Technology, Shizuoka University, 836 Ohya, Suruga-ku, Shizuoka 422-8529 Japan; 3 Graduate School of Pharmaceutical Sciences, Nagoya City University, 3-1 Tanabe-dori, Mizuho-ku, Nagoya 467-8603 Japan; 4 Medical & Biological Laboratories Co., Ltd., 4-5-3 Sakae, Naka-ku, Nagoya 460-0008 Japan; 5 Institute for Molecular Science and Okazaki Institute for Integrative Bioscience, National Institutes of Natural Sciences, 5-1 Higashiyama Myodaiji, Okazaki, 444-8787 Japan

## Abstract

Recombinant proteins produced in insect cells and insects, unlike those produced in mammalian cells, have pauci-mannose-type *N*-glycans. In this study, we examined complex-type *N*-glycans on recombinant proteins via coexpression of human β-1,2-*N*-acetylglucosaminyltransferase II (hGnT II) and human β1,4-galactosyltransferase (hGalT I) in silkworm pupae, by using the *Bombyx mori* nucleopolyhedrovirus (BmNPV) bacmid system. The actin A3 promoter from *B. mori* and the polyhedrin promoter from *Autographa californica* multiple nucleopolyhedroviruses (AcMNPVs) were used to coexpress hGnT II and hGalT I. These recombinant BmNPVs were coexpressed with human IgG (hIgG), hGnT II and hGalT I in silkworm pupae. When hIgG was coexpressed with hGnT II, approximately 15% of all *N*-glycans were biantennary, with both arms terminally modified with *N*-acetylglucosamine (GlcNAc). In contrast, when hIgG was coexpressed with both hGnT II and hGalT I under the control of the polyhedrin promoter, 27% of all *N*-glycans were biantennary and terminally modified with GlcNAc, with up to 5% carrying one galactose and 11% carrying two. The obtained *N*-glycan structure was dependent on the promoters used for coexpression of hGnT II or hGalT I. This is the first report of silkworm pupae producing a biantennary, terminally galactosylated *N*-glycan in a recombinant protein. These results suggest that silkworms can be used as alternatives to insect and mammalian hosts to produce recombinant glycoproteins with complex *N*-glycans.

## Introduction

Insect cell cultures have been widely used for the production of recombinant eukaryotic proteins^1^, because insect cells can post-translationally modify proteins with phosphates, *N*-glycans and *O*-glycans. These capabilities are superior to those of *Escherichia coli* for the expression of eukaryotic proteins. In addition, 2500 L-scale production of recombinant proteins has been performed in insect cell expression systems^2^. However, live insect larvae and pupae are also promising hosts as bioreactors for producing recombinant proteins, owing to their ability to be easily scaled up by using artificial diets^3, 4^.

The post-translational modifications that occur in insect cells are similar to those in mammalian cells; however, the *N*-glycans of proteins expressed in insect cells are mainly of the pauci-mannose type, whereas the *N*-glycans of proteins expressed in mammalian cells are of the complex type^5, 6^. This difference in *N*-glycans between these two cell types sometimes causes recombinant glycoproteins to be biologically inactive. The *N*-glycosylation of glycoproteins often affects their solubility, stability and bioactivity. In the case of therapeutic glycoproteins, *N*-glycosylation is critical for glycoprotein immunogenicity and clearance from serum in humans^7, 8^. In particular, sialic acids at the non-reducing ends of *N*-glycans on glycoproteins are important for prolonging their half-lives in serum. Therefore, most therapeutic glycoproteins are produced in mammalian cells, such as Chinese hamster ovary (CHO) cells^9^.

To improve the *N*-glycan patterns of recombinant glycoproteins expressed in insect cells, *Caenorhabditis elegans* β-1,2-*N*-acetylglucosaminyltransferase II (GnT II) and bovine β1,4-galactosyltransferase I (GalT I) have been coexpressed in insect cells using the Multibac system to achieve mammalian-like glycosylation in insect cells^10^. An insect cell line (SfSWT-1) that produces complex *N*-glycans containing sialic acid has been developed via the coexpression of five mammalian glycosyltransferases [human GnT I, human GnT II, bovine GalT I, rat α2,6-sialyltransferase I (ST6GalT I) and α2,3-sialyltransferase IV (ST3Gal IV)]^11^. To improve the rate of sialic acid addition to *N*-glycans in insect cells, insect cell lines that produce cytidine-5′-monophospho-*N*-acetylneuraminic acid (CMP-Neu5Ac) and efficiently transport it to the Golgi apparatus have also been developed^12–14^. Furthermore, *N*-acetylglucosaminidase (also known as fused lobes, FDL) plays the important role of producing pauci-mannose-type *N*-glycans for glycoproteins in insect cells^15, 16^. Suppression of FDL gene expression enhances the level of *N*-acetylglucosamine (GlcNAc)-terminated *N*-glycans in recombinant glycoproteins expressed in insect cells^17, 18^.

*Bombyx mori* (Silkworm) has been used for the production of recombinant proteins. Similarly to those in insect cells, most of the *N*-glycans on glycoproteins expressed in silkworms are of the pauci-mannose type^19, 20^. In the posterior silk gland (PSG), up to 17% of *N*-glycans are terminally galactosylated in transgenic silkworm larvae coexpressing human GnT II and bovine GalT I under the control of a PSG-specific promoter^21^. This result indicates that mammalian-like *N*-glycans can also be produced in the PSG of silkworms via the expression of mammalian glycosyltransferases. However, in this case, recombinant proteins must be expressed in the PSG of silkworms together with mammalian glycosyltransferases by using a transgenic technique, and generating transgenic silkworms to produce recombinant proteins is time consuming and labor intensive.

In this study, human GnT II (hGnT II) and human GalT I (hGalT I) were coexpressed with human immunoglobulin G (hIgG) in silkworm pupae by using recombinant *Bombyx mori* nucleopolyhedroviruses (BmNPVs) to produce galactosylated *N*-glycans on hIgG. The polyhedrin promoter from the *Autographa californica* multiple nucleopolyhedroviruses (AcMNPVs) and the actin A3 gene promoter from *B. mori* were used to express each human glycosyltransferase. Recombinant hIgG was purified from silkworm pupae, and its *N*-glycan modification was investigated. The expression system developed in this study is promising for the production of galactosylated recombinant proteins in silkworm pupae.

## Materials and Methods

### Recombinant BmNPV Bacmid Construction

The actin A3 promoter sequence from *B. mori* was amplified via PCR using the Actin-F and Actin-R primer set (Table 1). The polyhedrin promoter sequence in pFastBac1 (Thermo Scientific K. K., Yokohama, Japan) was replaced with each amplified DNA fragment by using InFusion technology (TAKARA Bio, Shiga, Japan). The resulting plasmid was designated pFast-Actin. The hGnT II and hGalT I genes were amplified via PCR using the hGnTII-F and hGnTII-R primers and the hGalTI-F and hGalTI-R primers, respectively. Each gene was inserted into pFast-Actin and pFastBac1. Next, pFast/P_Act_-hGnT II, pFast/P_Pol_-hGnT II, pFast/P_Act_-hGalT I and pFast/P_Pol_-hGalT I were constructed. Each plasmid was transformed into *E. coli* BmDH10Bac CP^−^Chi^−^^22^, and each recombinant BmNPV (BmNPV CP^−^Chi^−^/P_Act_-hGnT II, BmNPV CP^−^Chi^−^/P_Pol_-hGnT II, BmNPV CP^−^Chi^−^/P_Act_-hGalT I and BmNPV CP^−^Chi^−^/P_Pol_-hGalT I) bacmid was extracted from a transformed white colony. The BmNPV CP^−^Chi^−^/29IJ6 IgG bacmid was used to express hIgG in silkworm larvae^23^.

**Table 1:** Primers used in this study.

Name	Sequence (5′ to 3′)
Actin-F	CCGGAATATTAATAGAGGTACCACCACCCTGCC
Actin-R	CTTCGGACCGGGATCTCGATATCAAGCTTATCGATAC
pFast-F	GATCCCGGTCCGAAGCGCGCG
pFast-R	CTATTAATATTCCGGAGTACAC
hGnT II-F	CGGAATTCATGAGGTTCCGCATCTACAAAC
hGnT II-R	CGTCTAGACTACTGCAGTCTTCTATAACTTTTAC
hGalT I-F	CGGAATTCATGAGGCTTCGGGAGCCGCTC
hGalT I-R	CGTCTAGACTAGCTCGGTGTCCCGATGTC
BmIE-F	CCCGTAACGGACCTTGTGCTT
BmIE-R	TTATCGAGATTTATTTACATACAACAAG

### Protein Expression in Silkworm Pupae

To prepare infectious recombinant BmNPVs, each recombinant BmNPV bacmid was injected into silkworm larvae together with 1,2-dimyristyloxypropyl-3-dimethyl-hydroxyethyl ammonium bromide (DMRIE-C, Life Technologies Japan, Tokyo, Japan). Silkworm larvae were reared on an artificial diet, Silkmate 2 S (Nosan, Yokohama, Japan), for 6–7 days, and hemolymph was then collected from the silkworm larvae. This hemolymph was used to coexpress recombinant hIgG with human glycosyltransferases as an infectious recombinant BmNPV solution.

To coexpress hIgG and hGnT II, the hemolymph from a BmNPV CP^−^Chi^−^/29IJ6 IgG bacmid-injected silkworm larva was mixed with the hemolymph from either a BmNPV CP^−^Chi^−^/P_Act_-hGnT II bacmid- or BmNPV CP^−^Chi^−^/P_Pol_-hGnT II bacmid-injected silkworm larva. The hemolymph was diluted with phosphate-buffered saline (PBS, pH 7.4), and 5 × 10^5^ pfu of total recombinant BmNPVs was injected into silkworm pupae. The silkworm pupae were subsequently incubated for 4 days and were maintained at −80 °C before use.

### Determination of Recombinant BmNPV Titers

The recombinant BmNPV titers were determined through previously described methods^24^. The BmIE-F and BmIE-R primers were used for real-time PCR.

### SDS-PAGE, Western Blotting and Lectin Blotting

Proteins were separated via sodium dodecyl sulfate-polyacrylamide gel electrophoresis (SDS-PAGE) using 12% acrylamide gels and were subsequently subjected to western blotting. After SDS-PAGE, the proteins were blotted onto a polyvinylidene fluoride (PVDF) membrane with a Mini Trans-Blot Electrophoretic Transfer Cell (Bio-Rad, Hercules, CA, USA). For western blotting, after blocking with 5% skimmed milk in Tris-buffered saline containing 0.1% Tween 20 (TBST, pH 7.6), the membrane was incubated in either a 1,000-fold dilution of a rabbit polyclonal antibody against MGAT2 (hGnT II) (GeneTex, Los Angeles, CA, USA) or a 1,000-fold dilution of rabbit IgG B4GALT1 (hGalT I) (EnoGene Biotech, New York, NY, USA). The membrane was subsequently washed with TBST and then was incubated for 1 h in a 1:10,000 dilution of either an anti-mouse or anti-rabbit IgG antibody labeled with horseradish peroxidase (GE Healthcare Japan, Tokyo, Japan). Detection was performed by using ECL Plus western blotting reagent (GE Healthcare Japan). Specific bands were detected on a Fluor-S MAX MultiImager (Bio-Rad). For the lectin blots, the PVDF membrane was incubated with 2 μg/ml FITC-conjugated RCA120 (J-OIL MILLS, Tokyo, Japan), then washed with TBST. Specific fluorescent bands were detected with a Molecular Imager FX system (Bio-Rad).

### Purification of hIgG from Silkworm Pupae

Silkworm pupae were homogenized with TBST, and the homogenates were centrifuged (20,000 × *g*, 40 min) to remove insoluble materials. Recombinant human IgG was purified from the supernatant with Protein A Sepharose (GE Healthcare Japan), and 0.1 M citrate buffer (pH 3.0) was used to elute the recombinant human IgG. The pH of the eluted fractions was immediately adjusted to approximately pH 7.0 with 1 M Tris-HCl (pH 8.0). Finally, the purified hIgG was dialyzed with pure water and lyophilized for *N*-glycan analysis.

### Determination of *N*-Glycan Structure in Recombinant hIgG

All of the experimental procedures used for the determination of *N*-glycan structure, including the chromatographic conditions and glycosidase treatments, have been previously described^25–27^. Purified recombinant human IgG was proteolyzed with a mixture of chymotrypsin and trypsin and was further digested with glycoamidase A (Seikagaku Kogyo Co. Ltd, Tokyo, Japan) to release the *N*-glycans^28^. After removal of the peptides by using a carbon graphite column (300 mg, GL Science, Japan), the reducing ends of the *N*-glycans were derivatized with 2-aminopyridine (PA) (Wako Pure Chemical, Osaka, Japan). The mixture of the PA derivatives of the *N*-glycans was purified with cellulose columns (Sigma). The purified PA-glycan mixture was then applied to a Shim-pack HRC octadecyl silica (ODS) column (6.0-mm inner diameter: 150 mm; Shimadzu, Kyoto, Japan) and was collected as a fraction. Elution was performed at a flow rate of 1.0 ml/min at 55 °C with two solvents: A (10 mM sodium phosphate buffer, pH 3.8) and B (10 mM sodium phosphate buffer (pH 3.8) containing 0.5% 1-butanol). The gradient elution parameters were as follows: 0–60 min and a linear gradient of 20–50% solvent B. PA fluorescence was monitored at an excitation wavelength of 320 nm and an emission wavelength of 400 nm. For each peak, the elution time was recorded as a glucose unit (GU) value, on the basis of normalization to PA-derivatized isomalto-glycans with a polymerization degree of 421 residues. The ODS-separated fractions were subjected to matrix-assisted laser desorption ionization-time-of-flight-mass spectrometry (MALDI-TOF-MS) analysis^29^. The fractions were dried, diluted with pure water and (1 µL) spotted onto a target plate containing 1 µL of matrix solution (10 mg/mL of 2,5-dihydroxybenzoic acid (DHB) in 50% (v/v) acetonitrile in 0.1% TFA), and this was followed by MALDI-TOF-MS analysis using an AXIMA-CFR spectrometer (Shimadzu) operated in positive ion linear mode. The fractions that potentially contained two or more *N*-glycans were further separated with a TSK-gel Amide-80 column (4.6 mm i.d. ×250 mm, Tosoh, Tokyo, Japan). Elution was performed at a flow rate of 1.0 ml/min at 40 °C with two solvents: C (mixture of 65% (v/v) acetonitrile, 2.9% trimethylamine and 1.2% acetic acid, pH 7.3) and D (mixture of 50% (v/v) acetonitrile, 4.1% trimethylamine and 1.7% acetic acid, pH 7.3). The gradient elution parameters were as follows: 0–40 min and a linear gradient of 0–60% solvent D. The elution time of each peak was recorded as a GU value. The identification of *N*-glycan structure was based on the elution positions from these two types of columns compared with the PA-glycans in the GALAXY database^30^, followed by co-chromatography.

## Results

### Construction of Recombinant BmNPV Bacmids for the Expression of Human Glycosyltransferases in Silkworms

**Figure 1:**
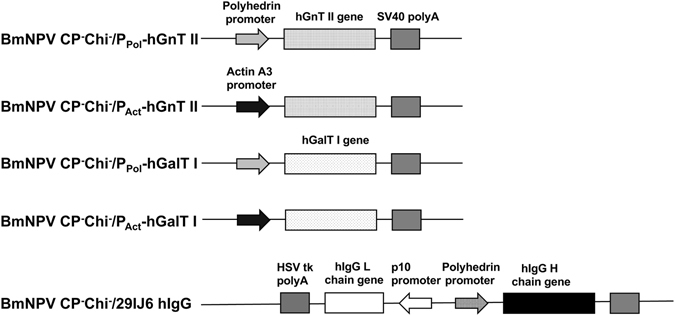
Expression cassettes in each recombinant BmNPV bacmid. Each recombinant BmNPV bacmid was constructed as described in the Materials and Methods section. Each BmNPV was prepared in silkworm larvae via the injection of each recombinant BmNPV bacmid into larvae.

In this study, the polyhedrin promoter from AcMNPV and the actin A3 promoter from *B. mori* were used to express human glycosyltransferases in silkworm pupae. pFastBac1 was used as a cloning vector to express human glycosyltransferases under the control of the polyhedrin promoter. To express human glycosyltransferases under the control of the A3 promoter, the polyhedrin promoter sequence of pFasBac1 was replaced with the actin A3 promoter from *B. mori*, and the generated products were designated pFast-Actin vectors. The hGnT II and hGalT I genes were inserted into the pFastBac1 and pFast-Actin vectors, respectively, downstream of each promoter. We used the constructed vectors, recombinant BmNPV (BmNPV CP^−^Chi^−^/P_Act_-hGnT II, BmNPV CP^−^Chi^−^/P_Pol_-hGnT II, BmNPV CP^−^Chi^−^/P_Act_-hGalT I and BmNPV CP^−^Chi^−^/P_Pol_-hGalT I) to construct bacmids (Fig. 1). To express hIgG, the BmNPV CP^−^Chi^−^/29IJ6 hIgG bacmid was used^23^. These recombinant BmNPV bacmids were injected into silkworm larvae individually, and the BmNPV titers in the hemolymph were measured. The harvested hemolymph was used as a recombinant BmNPV stock.

### Coexpression of hIgG with hGnT II in Silkworm Pupae

**Figure 2:**
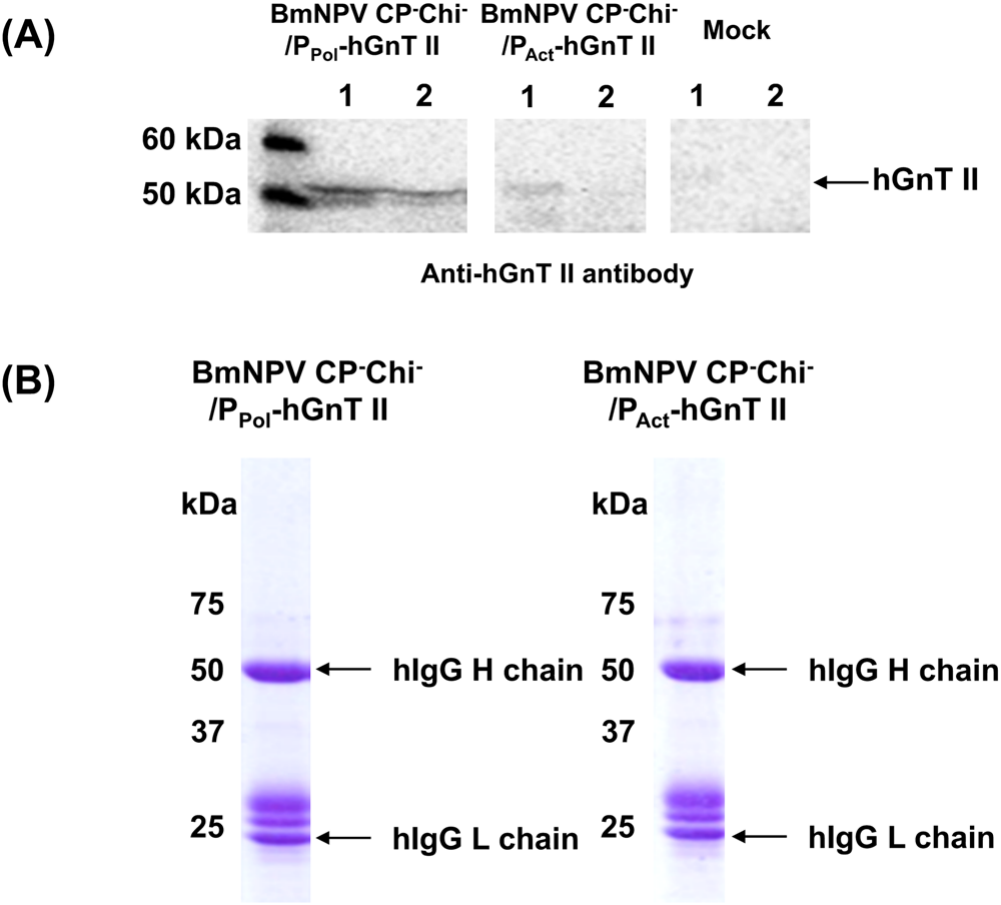
Expression of recombinant proteins in silkworm pupae with coexpression of hGnT II. (**A**) Expression of hGnT II using BmNPV CP^−^Chi^−^/P_Act_-hGnT II or BmNPV CP^−^Chi^−^/P_Pol_-hGnT II in silkworm pupae. Pupae were homogenized with TBST and centrifuged to separate the supernatant from the insoluble material. Western blotting was performed on both fractions. Lane 1: Supernatant, lane 2: Pellet. (**B**) SDS-PAGE with CBB staining using hIgG purified from BmNPV CP^−^Chi^−^/29IJ6 hIgG-injected silkworm pupae. The left and right panels show the proteins that were coexpressed with hGnT II under the control of either the polyhedrin (P_pol_-hGnT II) or actin A3 promoter (P_Act_-hGnT II).

The expression of hGnT II in silkworm pupae was achieved using either BmNPV CP^−^Chi^−^/P_Act_-hGnT II or BmNPV CP^−^Chi^−^/P_Pol_-hGnT II and was analyzed through western blotting (Fig. 2A and Fig. S1). Specific bands were detected at approximately 50 kDa; however, this molecular weight was higher than that estimated on the basis of the amino acid sequence (48 kDa), because hGnT II has two putative *N*-glycosylation sites. No bands were detected in the mock pupae. The expression level of hGnT II under the control of the polyhedrin promoter was higher than that under the control of the actin A3 promoter in silkworm pupae.

To coexpress hIgG with hGnT II in silkworm pupae, BmNPV CP^−^Chi^−^/29IJ6 IgG and each recombinant BmNPV (BmNPV CP^−^Chi^−^/P_Act_-hGnT II and BmNPV CP^−^Chi^−^/P_Pol_-hGnT II) were injected into silkworm pupae at a 1:1 ratio, on the basis of the number of pfu (total 5 × 10^5^ pfu/pupa). The recombinant hIgG coexpressed with hGnT II was purified via Protein A Sepharose column chromatography. The hIgG H and L chains were detected, but protein bands between the hIgG H and L chains were also observed (Fig. 2B and Fig. S2). These bands appeared to be degraded H chains, because the recombinant hIgG purified from the hemolymph contained some proteins for which the antibody reacted with human IgG^23^. Therefore, this purified hIgG was used for *N*-glycan structure analysis.

**Figure 3:**
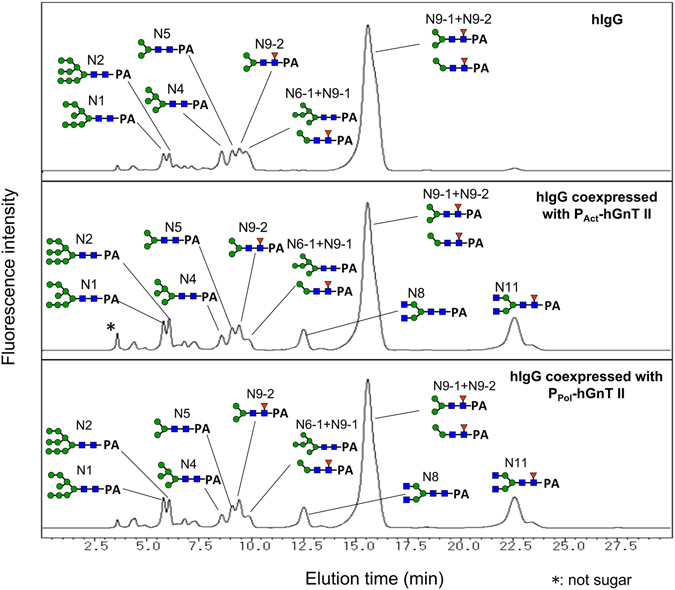
Chromatograms of PA-derivatized *N*-glycans derived from recombinant IgG coexpressed with hGnT II in silkworms, as determined via ODS column chromatography. N1 to N11 are the numbers of *N*-glycans, as shown in Fig. 4.

**Figure 4:**
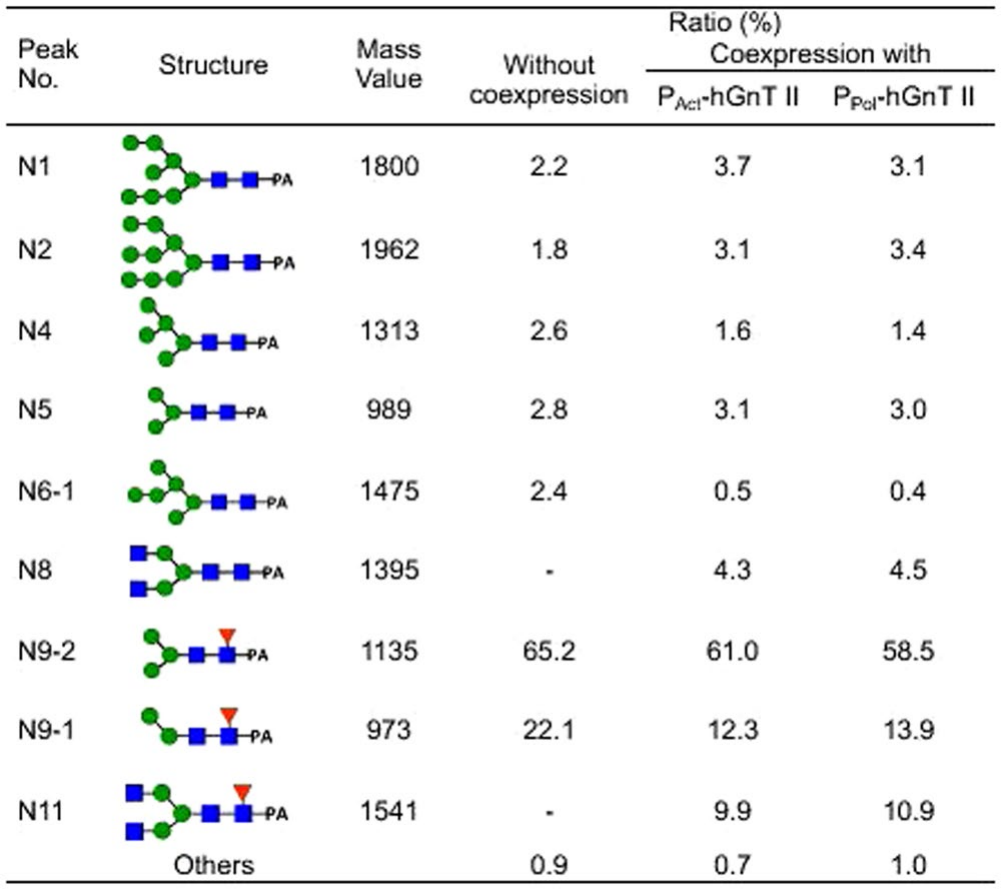
*N*-glycan structures of hIgG coexpressed with hGnT II in silkworm pupae.

*N*-glycans were cleaved from purified hIgG and derivatized with 2-aminopyridine (PA). The PA-*N*-glycans were separated via ODS column chromatography and amide column chromatography. The ODS column chromatograms are shown in Fig. 3. When hIgG was coexpressed with hGnT II, a new peak (N8) that was not observed without the coexpression of hGnT II appeared. The detailed *N*-glycan structures from the purified IgG are shown in Fig. 4. Terminal GlcNAc residues were not observed in the *N*-glycans of hIgG that were purified from silkworm pupae. However, when hIgG was coexpressed with hGnT II under the control of the actin A3 promoter (P_Act_-hGnT II) and with hGnT II under the control of the polyhedrin promoter (P_Pol_-hGnT), 14.2% and 15.4% of the *N*-glycans, respectively, were GlcNAc terminated (N8: GlcNAc_2_Man_3_GlcNAc_2_, N11: GlcNAc_2_Man_3_GlcNAc(Fuc)GlcNAc). This result indicated that the expressed GnT II modified the *N*-glycans of hIgG and produced GlcNAc-terminated *N*-glycans in silkworm pupae.

### Coexpression of hIgG with both hGnT II and hGalT I in Silkworm Pupae

**Figure 5:**
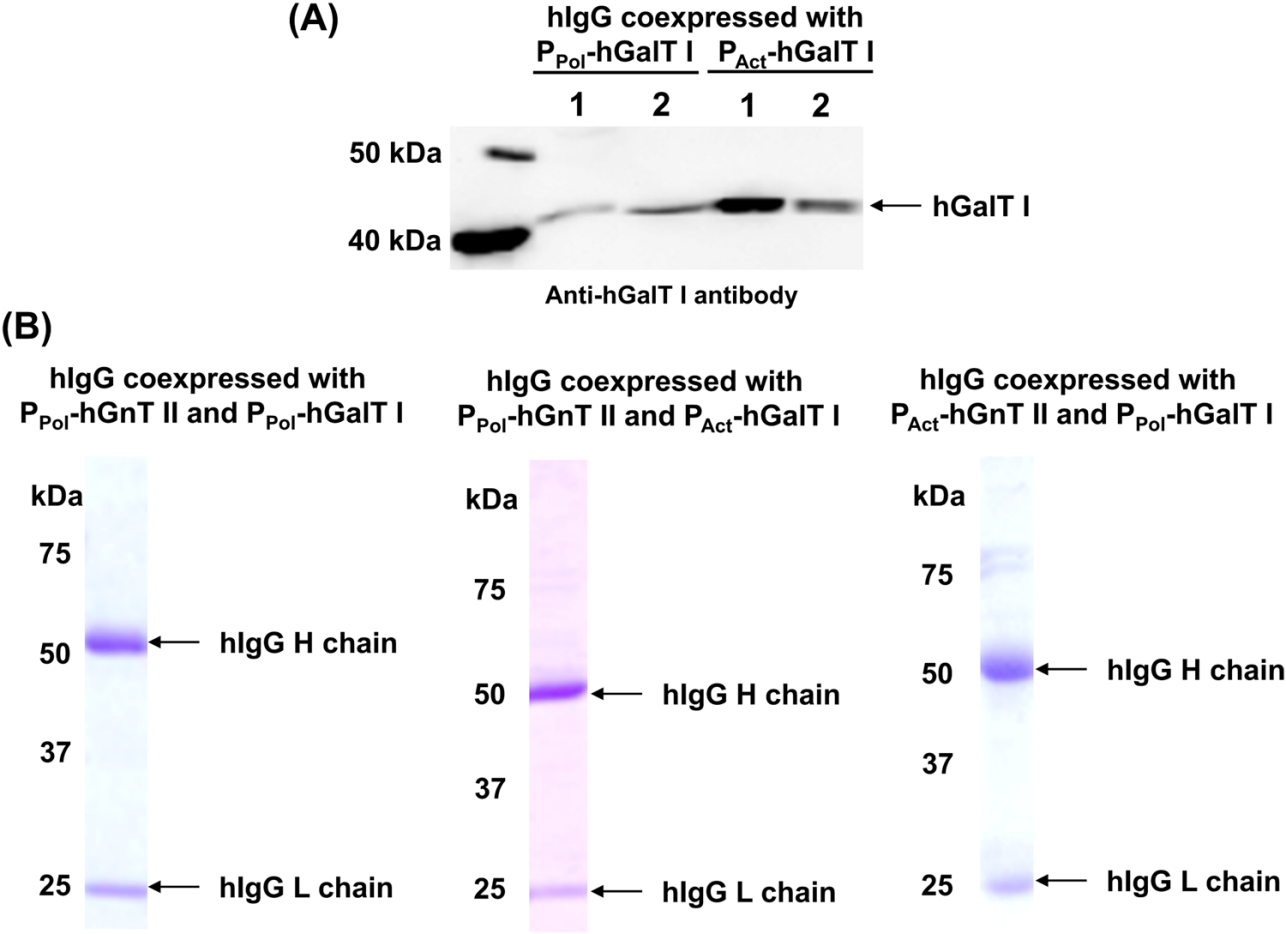
Expression of recombinant proteins in silkworm pupae with coexpression of hGnT II and hGalT I. (**A**) Expression of hGalT I in silkworm pupae by using BmNPV CP^−^Chi^−^/P_Act_-hGalT I or BmNPV CP^−^Chi^−^/P_Pol_-hGalT I. Pupae were homogenized with TBST and centrifuged to separate the supernatant from the insoluble material. Western blotting was performed on both fractions. Lane 1: Supernatant, lane 2: Pellet. (**B**) SDS-PAGE of purified hIgG coexpressed with P_Pol_-hGnT II and P_Pol_-hGal T I (left panel), P_Pol_-hGnT II and P_Act_-hGal T I (middle panel) and P_Act_-hGnT II and P_Pol_-hGal T I (right panel). Gels were stained with CBB.

The expression of hGalT I in silkworm pupae by using BmNPV CP^−^Chi^−^/P_Act_-hGalT I or BmNPV CP^−^Chi^−^/P_Pol_-hGalT I was expressed as judged by Western blotting (Fig. 5A and Fig. S3). Specific bands were detected in the supernatants and pellets from pupae extracts at 40 kDa (estimate molecular weight: 39 kDa), thus indicating that each recombinant BmNPV facilitated the expression of hGalT I in silkworm pupae.

To terminally galactosylate the *N*-glycans of hIgG in silkworm pupae, hIgG was coexpressed with hGnT II and hGalT I in the following combinations: hGnT II under the control of the actin A3 promoter (P_Act_-hGnT II) and hGalT I under the control of the polyhedrin promoter (P_Pol_-hGalT I); hGnT II under the control of the polyhedrin promoter (P_Pol_-hGnT II) and hGalT I under the control of the actin A3 promoter (P_Act_-hGalT I); and hGnT II under the control of the polyhedrin promoter (P_Pol_-hGnT II) and hGalT I under the control of the polyhedrin promoter (P_Pol_-hGalT I). Three recombinant BmNPVs were injected into silkworm pupae at a 1:3:3 ratio (IgG:hGnT II:hGalT I, total 5 × 10^5^ pfu/pupa). The resulting hIgG expressed in the pupae was purified via Protein A Sepharose column chromatography. The purified H and L chains of hIgG were confirmed in every combination (Fig. 5B and Fig. S4).

The terminal galactose residues in the *N*-glycans of hIgG were confirmed through lectin blot analysis using FITC-conjugated RCA120. A specific band of hIgG was observed under either P_Act_-hGnT II and P_Pol_-hGalT I coexpression (Lane 5 in Fig. S5) or P_Pol_-hGnT II and P_Pol_-hGalT I coexpression (Lane 6 in Fig. S5). However, no bands were observed when P_Pol_-hGnT II and P_Act_-hGalT I were coexpressed with hIgG, although hGalT I was expressed (Lane 4 in Fig. S5). This finding indicates that the combination of P_Pol_-hGnT II and P_Act_-hGalT I resulted in no galactosylation in silkworm pupae.

**Figure 6:**
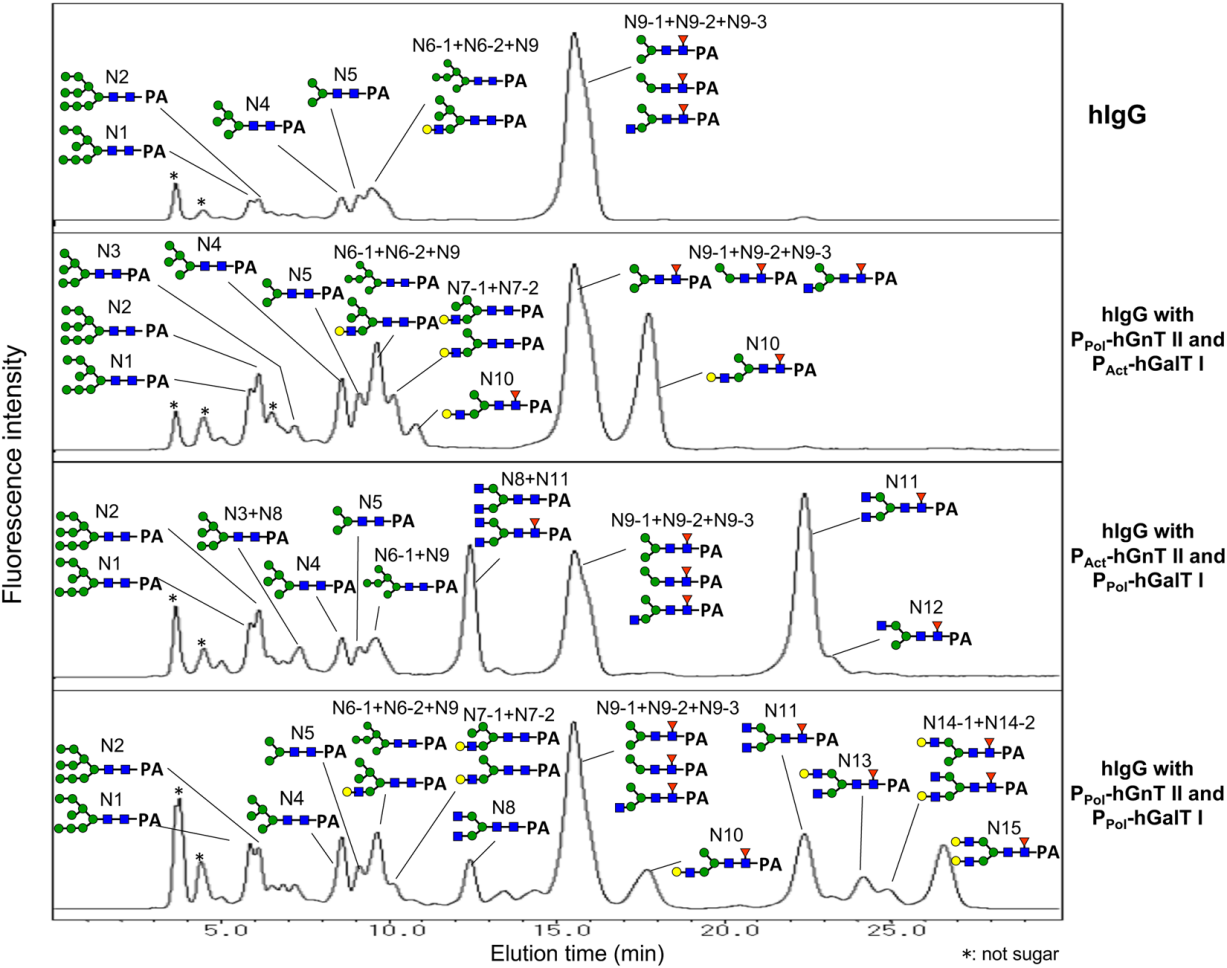
Chromatograms of PA-derivatized *N*-glycans derived from recombinant IgG from silkworm coexpressed with hGnT II and hGalT I, as determined via ODS column chromatography. N1 to N15 correspond to the number of *N*-glycans, as shown in Fig. 7.

**Figure 7:**
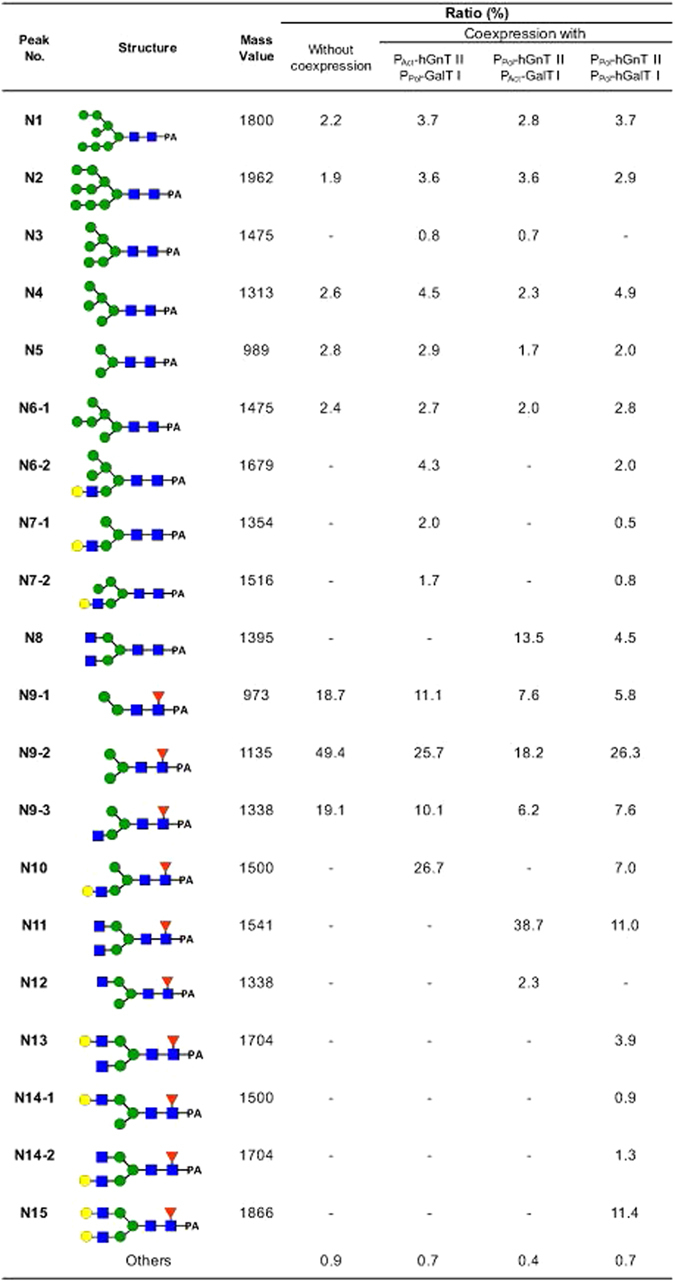
*N*-glycan structures of hIgG coexpressed with hGnT II and hGalT I in silkworm pupae.

For detailed analysis of the *N*-glycan structures, the PA-*N*-glycans prepared from each recombinant IgG were separated via ODS column chromatography and amide column chromatography. Compared with the *N*-glycans of the hIgG from silkworm pupae, in the *N*-glycans from pupae in which hIgG was coexpressed with hGnT II and hGalT I, new peaks (N10–N15) were observed at later retention times (Fig. 6). However, the chromatograms of the *N*-glycans from hIgG coexpressed with the three combinations of hGnT II and hGalT I were different from one another. The detailed *N*-glycan structures were determined and are shown in Fig. 7. Under the combination of P_Act_-hGnT II and P_Pol_-hGalT I, GalGlcNAcMan_3_GlcNAc(Fuc)GlcNAc (N10) was observed at a rate of 26.7%, but biantennary galactosylated *N*-glycans were not observed. In contrast, for the combination of P_Pol_-hGnT II and P_Pol_-hGalT I, a biantennary galactosylated *N*-glycan (N15: Gal_2_ GlcNAc_2_Man_3_GlcNAc(Fuc)GlcNAc) was detected at a rate of 11.4%. Several mono-galactosylated *N*-glycans (N6-2: GalGlcNAcMan_5_GlcNAc_2_, N7-1: GalGlcNAcMan_4_GlcNAc_2_; N10: GalGlcNAcMan_3_GlcNAc(Fuc)GlcNAc, N13: GalGlcNAc_2_Man_3_GlcNAc(Fuc)GlcNAc, N14-1: GalGlcNAcMan_3_GlcNAc(Fuc)GlcNAc, N14-2: GalGlcNAc_2_Man_3_GlcNAc(Fuc)GlcNAc) were also observed. For the combination of P_Pol_-hGnT II and P_Act_-hGalT I, no terminally galactosylated *N*-glycans were identified, in agreement with the results of lectin blotting (Fig. S5). Interestingly, 52.2% of the *N*-glycans in this combination exhibited biantennary terminal GlcNAc residues (N11: GlcNAc_2_Man_3_GlcNAc(Fuc)GlcNAc, N8: GlcNAc_2_Man_3_GlcNAc_2_). These results indicated that the polyhedrin promoter is suitable for the coexpression of both hGnT II and hGalT I to produce biantennary galactosylated *N*-glycans in silkworm pupae. However, pauci-mannose-type and high-mannose-type *N*-glycans were still observed at rates of 34.1% and 14.3%, respectively, under the combination of P_pol_-hGnT II and P_pol_-hGalT I. In Fig. 7, in contrast to the structures shown in Fig. 4, a mono-terminally GlcNAcated *N*-glycan (N9-3: GlcNAcMan_3_GlcNAc(Fuc)GlcNAc) was observed, even when the pupae were infected with only BmNPV CP^−^Chi^−^/29IJ6 hIgG. Pauci- and oligomannosidic glycans were often produced on recombinant hIgG expressed in silkworm pupae without the coexpression of human glycosyltransferases. This *N*-glycan is sometimes observed in expressed recombinant proteins in insects^6, 19, 31^. This result suggests that silkworm pupae are capable of producing complex *N*-glycans on recombinant hIgG, albeit at somewhat lower levels than those in other systems.

## Discussion

In this study, hIgG was coexpressed with hGnT II and hGalT I in silkworm pupae to produce biantennary terminally galactosylated *N*-glycans. Two promoters, the actin A3 and polyhedrin promoters, were used to express hGnT II and hGalT I. The polyhedrin promoter is active during the late stage of baculoviral infection and has been used to express mammalian glycosyltransferases for the engineering of *N*-glycans in insect cells^10^. The actin promoter from *B. mori* is a constitutive promoter and has also been used for protein expression in insect cells^32, 33^. Only coexpression of P_Pol_-hGnT II and P_Pol_-hGalT I led to the expression of up to 35% biantennary terminally galactosylated *N*-glycans in silkworm pupae (Fig. 7). In contrast, the coexpression of P_Pol_-hGnT II and P_Act_-hGalT I primarily produced biantennary terminally GlcNAcated *N*-glycan. In addition, the expression of P_Act_-hGnT II and P_Pol_-hGalT I produced only mono-galactosylated *N*-glycans. Overall, the data indicated that using the actin A3 promoter for one or both glycosyltransferases rather than the polyhedrin promoter led to a lack of expression in the Golgi; thus, although remodeling was achieved when hGnTII was expressed under the control of the actin A3 promoter, the use of this promoter is not recommended.

Glycosyltransferases sequentially localize to the endoplasmic reticulum and Golgi, thus allowing for correct *N*-glycan modification^34^. In addition, some glycosyltransferases form hetero-complexes with other glycosyltransferases in the Golgi, thus allowing for efficient and sequential *N*-glycan modification^35, 36^. Overexpression of proteins in the Golgi often causes the expressed proteins to be mislocalized in cells^37^. Glycosyltransferase overexpression may disturb the sequence of the glycosyltransferases in the Golgi required for correct *N*-glycan modification. This possibility suggests that the choice of promoters for coexpressing several glycosyltransferases may be crucial for achieving the correct *N*-glycan modification in the Golgi. In particular, the polyhedrin promoter is active during the very late stages of baculoviral infection in which the protein secretory pathway is disturbed^38^. Additional investigation of the promoters used for the expression of glycosyltransferases are necessary to improve the efficiency of the production of galactosylated recombinant proteins in silkworms by using recombinant BmNPVs.

As shown in Fig. 7, 46.4% of the *N*-glycans on hIgG remained pauci-mannose- and high-mannose-type *N*-glycans, even when hGnT II and hGalT I were coexpressed. In a previous study, engineered bovine GalT I has been shown to greatly enhance the production of biantennary terminally galactosylated *N*-glycans^39^. This engineered bovine GalT I has one mutation at ^282^Leu and contains the cytoplasmic/transmembrane/stem (CTS) domains of human α1,3-fucosyltransferase 7 (FUT7) instead of its native CTS domain. The CTS domains of human FUT7 retain bovine GalT I in the Golgi, which catalyzes sequential *N*-glycan modification. The CTS domains of glycosyltransferases are critical for their localization in the Golgi and for sequential *N*-glycan modification. In yeast, the use of optimal CTS domains and orthologous genes from other species may be required for the expression of glycosyltransferases and glycosidases to produce biantennary terminally GlcNAcated *N*-glycans^40^. In silkworms as well as in yeast, replacement of the CTS domains of glycosyltransferases (hGnT II and hGalT I) with optimal CTS regions or the use of orthologous genes from other species may be required for the efficient modification of *N*-glycans.

To coexpress three proteins (hIgG, hGnT II and hGalT I) in silkworm pupae, three recombinant BmNPVs were simultaneously injected into silkworm pupae. Two strategies for expressing several proteins using recombinant baculoviruses have been reported^41^. One strategy is to coexpress several proteins by using a recombinant baculovirus that contains several gene expression cassettes. Another strategy is the coinfection of several mono-cistronic recombinant baculoviruses, which we used in this study to express hIgG, hGnT II and hGalT I in silkworm pupae. To express recombinant proteins with auxiliary proteins, such as chaperones, coinfection systems are generally adopted^41^. However, in the case of coinfection systems for the coexpression of several proteins, optimization of the infection conditions, the M.O.I., the proportion of each recombinant baculovirus, the injection times and the choice of promoter are required for the efficient expression of recombinant proteins in insect cells^41–44^.

Galactosylated *N*-glycans of silk gland proteins have been produced in the PSG of transgenic silkworms coexpressing human GnT II and bovine GalT I under the control of a PSG-specific promoter^21^. To produce recombinant proteins in the PSG of this transgenic silkworm expressing mammalian glycosyltransferases, the corresponding gene must be transgenically inserted into the genomes of transgenic silkworms. This expression system cannot achieve rapid production of galactosylated recombinant proteins in silkworms. In contrast, in this study, recombinant BmNPVs expressing human glycosyltransferases were shown to facilitate the rapid production of galactosylated recombinant proteins in silkworm larvae and pupae via coinfection with multiple recombinant BmNPV infections. It is highly advantageous to produce recombinant glycoproteins with attached complex-type *N*-glycans.

## Electronic supplementary material

Supplementary information
