# Supplementary material for: N-Glycan Modification of a Recombinant Protein via Coexpression of Human Glycosyltransferases in Silkworm Pupae
Source: Sci Rep. 2017 May 3;7:1409. doi: 10.1038/s41598-017-01630-6 (PMC5431099; doi:10.1038/s41598-017-01630-6)
Supplement: Supplementary file 1 — Supplementary information [file 41598_2017_1630_MOESM1_ESM.pdf]

Supplementary Information

N-Glycan Modification of a Recombinant Protein via Coexpression of Human Glycosyltransferases in Silkworm Pupae

Tatsuya Kato[a,b], Natsumi Kako[a], Kotaro Kikuta[a], Takatsugu Miyazaki[a,b], Sachiko Kondo[c,d], Hirokazu Yagi[c], Koichi Kato[c,d,e] & Enoch Y. Park[a,b*]

E-mails:
kato.tatsuya@shizuoka.ac.jp (TK)
szd_723@yahoo.co.jp (NK)
si0122ho@yahoo.co.jp (KK)
miyazaki.takatsugu@shizuoka.ac.jp (TM)
kondo050525@yahoo.co.jp (SK)
hyagi@phar.nagoya-cu.ac.jp (HY)
kkato@phar.nagoya-cu.ac.jp (KK)
park.enoch@shizuoka.ac.jp (EYP)

* Corresponding authors. *E-mail address:* park.enoch@shizuoka.ac.jp (EYP)
[a] *Laboratory of Biotechnology, Department of Applied Biological Chemistry, Faculty of Agriculture, Shizuoka University, 836 Ohya, Suruga-ku, Shizuoka 422-8529, Japan*
[b] *Laboratory of Biotechnology, Research Institute of Green Science and Technology, Shizuoka University, 836 Ohya, Suruga-ku, Shizuoka 422-8529, Japan; Telephone & Fax: +81-54-238-4887*
[c] *Graduate School of Pharmaceutical Sciences, Nagoya City University, 3-1 Tanabe-dori, Mizuho-ku, Nagoya 467-8603, Japan*
[d] *Medical & Biological Laboratories Co., Ltd., 4-5-3 Sakae, Naka-ku, Nagoya 460-0008 Japan*
[e] *Institute for Molecular Science and Okazaki Institute for Integrative Bioscience, National Institutes of Natural Sciences, 5-1 Higashiyama Myodaiji, Okazaki 444-8787, Japan*

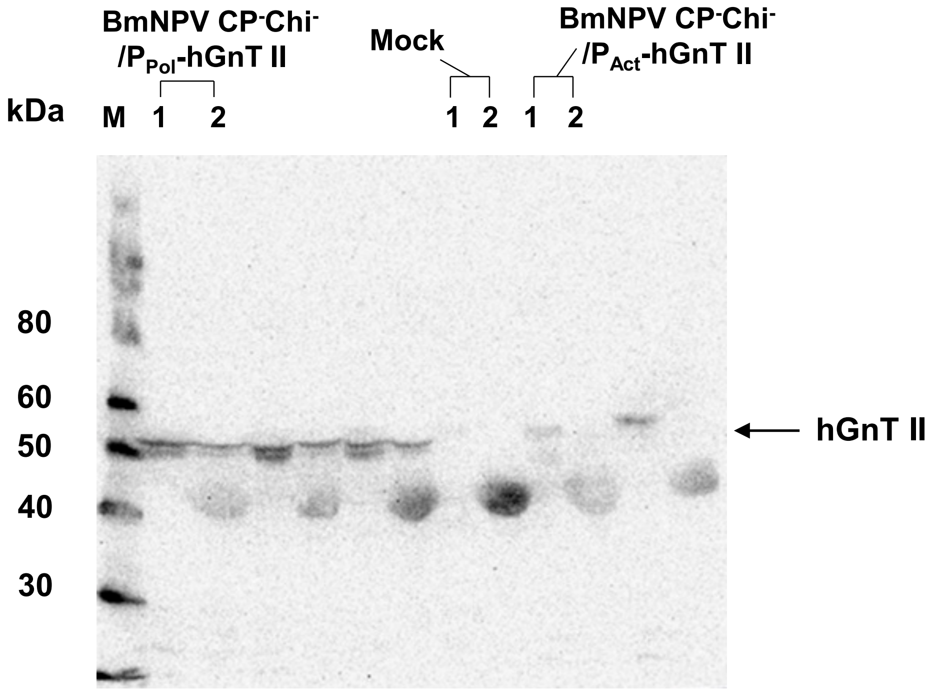

Figure S1. The western blotting data of Fig 2 (A). Lane 1: Supernatant, lane 2: Pellet. The band observed at 40 kDa is non-specific because the band was also observed in the pellet fraction in the mock sample.

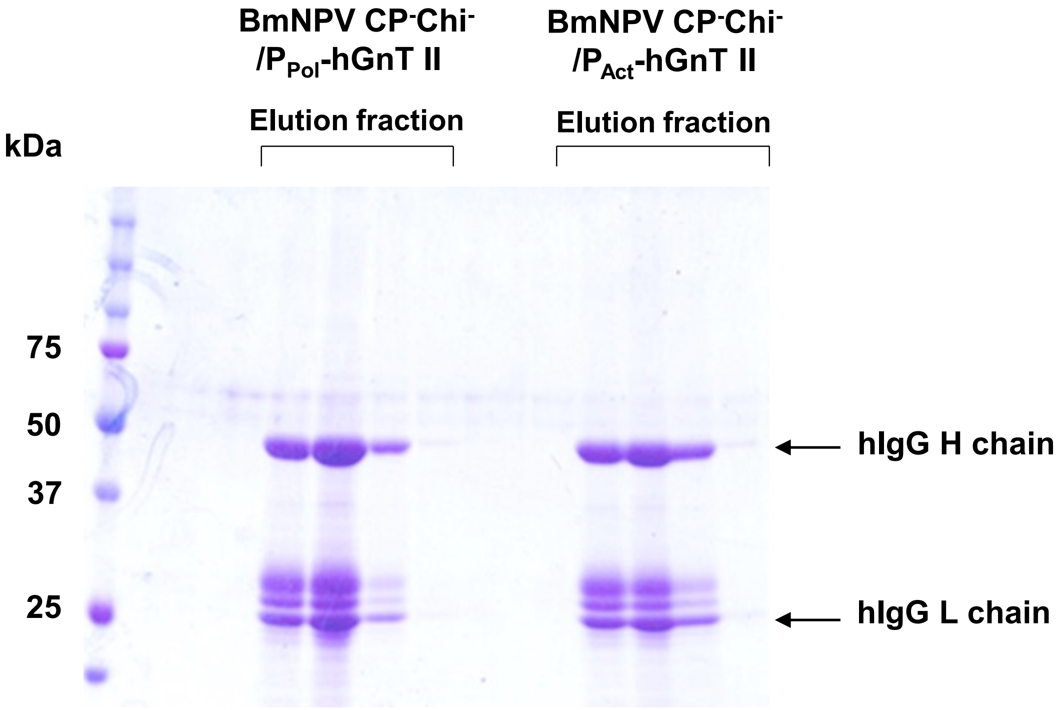

Figure S2. The western blotting data of Fig 2 (B).

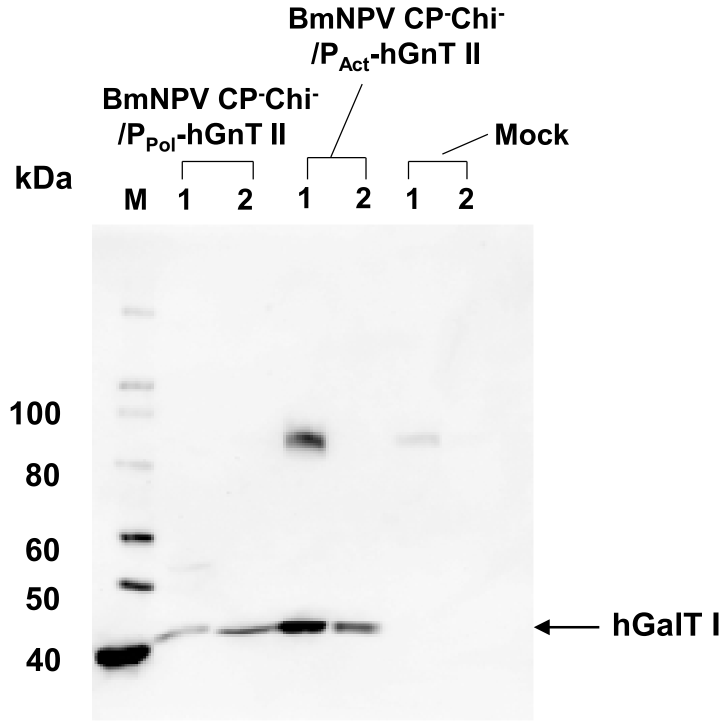

Figure S3. The western blotting data of Fig 4 (A). Lane 1: Supernatant, lane 2: Pellet. The band observed at 90 kDa is non-specific because the band was also observed in the supernatant fraction in the mock sample.

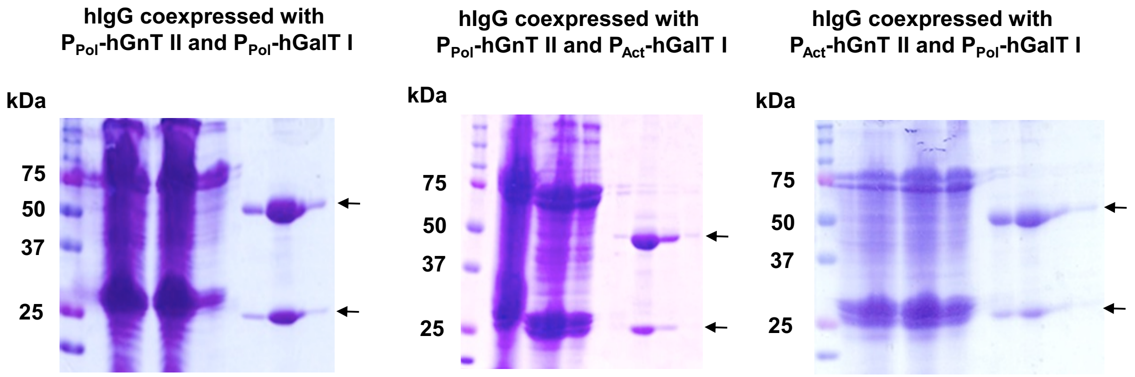

Figure S4. The western blotting data of Fig 4 (B). Arrows indicate hIgG H chain (at around 50 kDa) and light chain (at around 25 kDa).

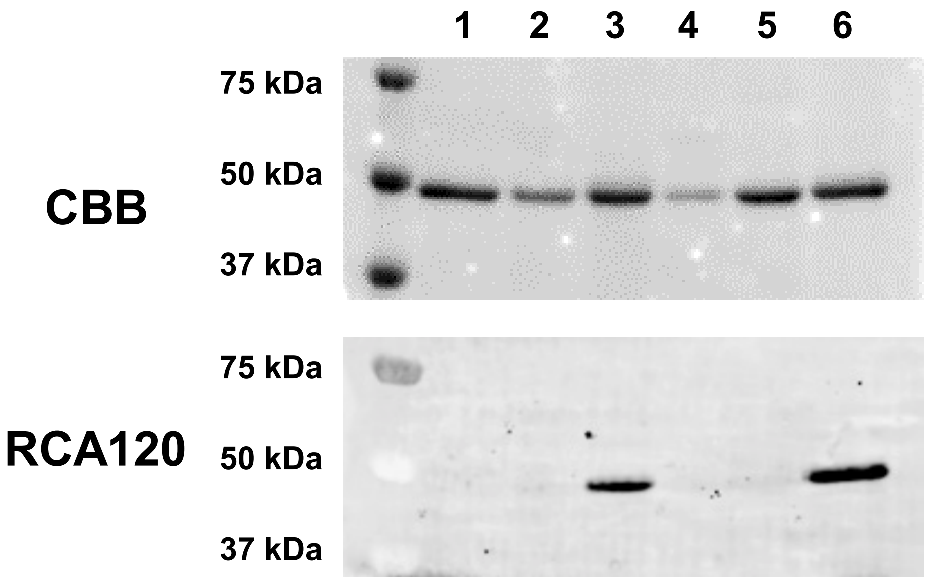

Figure S5. Lectin blot analysis of purified hIgG using FITC-conjugated RCA120. In this lectin blot, 10 μg of each purified hIgG was used. Lane 1: hIgG, lane 2: hIgG coexpressed with P_{Act}-hGnT II, lane 3: hIgG coexpressed with P_{Act}-hGnT II and P_{Pol}-hGalT I, lane 4: hIgG coexpressed with P_{Pol}-hGnT II, lane 5: hIgG coexpressed with P_{Pol}-hGnT II and P_{Act}-hGalT I, lane 6: hIgG coexpressed with P_{Pol}-hGnT II and P_{Pol}-hGalT I.
